# Reliability of the performance-based measure of executive functions in people with schizophrenia

**DOI:** 10.1186/s12888-021-03562-y

**Published:** 2021-11-10

**Authors:** En-Chi Chiu, Ya-Chen Lee, Shu-Chun Lee, I-Ping Hsueh

**Affiliations:** 1 Department of Long-Term Care, National Taipei University of Nursing and Health Sciences, Taipei, Taiwan; 2 Department of Occupational Therapy, Taipei City Psychiatric Center, Taipei City Hospital, Taipei, Taiwan; 3 Department of Occupational Therapy, College of Medical and Health Science, Asia University, Taichung, Taiwan; 4 Department of Occupational Therapy, I-Shou University, Kaohsiung, Taiwan; 5 University of Taipei, Taipei, Taiwan; 6 School of Occupational Therapy, College of Medicine, National Taiwan University, No.17, Xuzhou Rd., Zhongzheng Dist, Taipei City, Taiwan; 7 Department of Physical Medicine and Rehabilitation, National Taiwan University Hospital, Taipei, Taiwan

**Keywords:** Executive functions, Schizophrenia, Occupational therapy, Psychometric evaluation, Reliability

## Abstract

**Background:**

The Performance-based measure of Executive Functions (PEF) with four domains is designed to assess executive functions in people with schizophrenia. The purpose of this study was to examine the test-retest reliability of the PEF administered by the same rater (intra-rater agreement) and by different raters (inter-rater agreement) in people with schizophrenia and to estimate the values of minimal detectable change (MDC) and MDC%.

**Methods:**

Two convenience samples (each sample, *n* = 60) with schizophrenia were conducted two assessments (two weeks apart). The intraclass correlation coefficient (ICC) was analyzed to examine intra-rater and inter-rater agreements of the test-retest reliability of the PEF. The MDC was calculated through standard error of measurement.

**Results:**

For the intra-rater agreement study, the ICC values of the four domains were 0.88–0.92. The MDC (MDC%) of the four domains (volition, planning, purposive action, and perfromance effective) were 13.0 (13.0%), 12.2 (16.4%), 16.2 (16.2%), and 16.3 (18.8%), respectively. For the inter-rater agreement study, the ICC values of the four domains were 0.82–0.89. The MDC (MDC%) were 15.8 (15.8%), 17.4 (20.0%), 20.9 (20.9%), and 18.6 (18.6%) for the volition, planning, purposive action, and performance effective domains, respectively.

**Conclusions:**

The PEF has good test-retest reliability, including intra-rater and inter-rater agreements, for people with schizophrenia. Clinicians and researchers can use the MDC values to verify whether an individual with schizophrenia shows any real change (improvement or deterioration) between repeated PEF assessments by the same or different raters.

**Supplementary Information:**

The online version contains supplementary material available at 10.1186/s12888-021-03562-y.

## Background

Executive dysfunction is one of the major cognitive dysfunction in people with schizophrenia. Executive functions indicate higher-level cognitive functions that enable people to form goals, plan how to achieve those goals, and then execute the plans effectively [1–3]. According to recent evidences [4–6], around 50–70% of people with schizophrenia who have executive dysfunction show difficulty in planning, mental flexibility, and problem solving, which affect their independence in the activities of daily living, work, and social participation at home and in the community [3, 7, 8]. Therefore, a reliable measure of executive functions for people with schizophrenia is a necessity for clinicians to assess their difficulties of executive functions and to make treatment plans.

The Performance-based measure of Executive Functions (PEF) is an executive function measure designed for people with schizophrenia [9], and it offers three merits. First, as a theory-based measure, it was developed based on the Lezak model, which is one of the commonly recommended theories of executive functions and conceptualizes executive functions as four domains (volition, planning, purposive action, and effective performance) [9, 10]. Volition domain is the ability to form goals. Planning is the ability to recognize and organize materials or steps for accomplishing a goal. Purposive action means the ability to start, continue, shift, and stop steps of the planned actions. Effective performance is the ability to monitor, self-correct, and regulate performance quality [11]. Second, the PEF items were created using instrumental activities of daily living that people with schizophrenia felt were difficult to perform. Executive functions are concerned with doing non-routine activities in an non-automated manner [12]. The instrumental activities of daily living are not performed habitually and people with schizophrenia cannot perform the difficult instrumental activities of daily living spontaneously. Third, the raw ordinal scores of the four PEF domains can be transformed into Rasch interval scores [9]. As previous study has proposed that the PEF fits the assumptions of the Rasch model, the ordinal scale of the PEF could be transformed into an interval-level scale [9]. An interval scores presents equal values between any two points on the scale [13]. The interval property supports arithmetic operations and analysis by parametric statistics [14]. It has been reported in literatures that parametric tests are in general more powerful than nonparametric tests [15, 16]. Therefore, the PEF has good potential for clinicians and researchers to identify the status of executive functions in four domains for people with schizophrenia.

Construct validity (unidimensionality of each domain) and Rasch reliability in the PEF has been evaluated in people with schizophrenia [9]. However, test-retest reliability of the PEF has not been evaluated, limiting its applicability in clinical and research settings. For a performance-based measure with observational ratings, test-retest reliability is concerned with the level of consistency when repeating the same test on the same subject at different points in time [17]. The level of consistency could be examined by the agreement between two test sessions conducted by the same rater (intra-rater agreement) or by different raters (inter-rater agreement). The importance of achieving consistency among ratings is to verify measurement errors. The measurement errors can cause inaccurate estimates of participants’ performance in clinical and research settings. Minimal detectable change (MDC) is defined as the minimal change measurable besides random measurement error with a particular confidence level between repeated assessments [18]. The MDC can be employed to determine whether a real change is achieved between adjacent assessments by the same or different raters. It is necessary for a performance-based measure to have satisfactory test-retest reliability to ensure consistent results between repeated assessments administered by the same or different raters. Therefore, the purpose of this study was to examine the test-retest reliability of the PEF by the same rater (intra-rater agreement) and by different raters (inter-rater agreement) in people with schizophrenia and to estimate the values of MDC and MDC% of each domain. The MDC values can be employed to determine whether a real change is achieved between adjacent assessments by the same or different raters.

## Materials and methods

### Participants

We recruited two convenience samples from one psychiatric center in northern Taiwan between April to June 2014. One sample was recruited for the intra-rater agreement study and the other was for the inter-rater agreement study. People with schizophrenia were randomly grouped into two convenience samples. The inclusion criteria for the patients were: (1) diagnosis of schizophrenia based on the Diagnostic and Statistical Manual of Mental Disorders, 5th edition; (2) aged over 20 years; (3) onset for > 2 years; (4) stable and consistent dose of antipsychotic medication received for at least 3 months; and (5) willing to sign the informed consent. The exclusion criteria for the patients were: (1) history of severe brain injury; (2) diagnosis of substance abuse; and (3) diagnosis of intellectual developmental disorder. This study was accepted by the institutional review board in the local hospital.

A sample size of 55 participants was calculated for a reliability study with an intraclass correlation coefficient (ICC) of 0.80 at a significance level of 0.05 [19]. Thus, we decided to recruit 60 people with schizophrenia for the intra-rater agreement study and inter-rater agreement study.

### Procedure

Before the study, rater A (the PEF developer) gave rater B training to administer the PEF. During the training, rater A observed rater B testing three people without schizophrenia and confirmed that rater B explained the instructions correctly and fluently, manipulated the assessment tools appropriately, and understood the scoring standards. Then, the PEF was administered on a small sample size (ten people with schizophrenia) by rater B. Rater A observed the assessments and gave PEF scores simultaneously to ensure proper administration procedures and scoring. When the study started, the PEF ratings were independent. Neither raters discussed the ratings with each other to avoid affecting the reliability. People in the two convenience samples who met the inclusion criteria were administered with the PEF face-to-face, one-on-one, twice over a 2-week interval in a quiet environment. We chose a 2-week interval based on the stability of psychopathological characteristics for people with schizophrenia [20]. Each assessment lasted about 50 min. For the intra-rater agreement study, rater A administered the PEF twice. For the inter-rater agreement study, rater A performed the first assessment and rater B performed the second assessment. If a patient’s psychiatric drugs and doses had been adjusted in-between the study periods, then the second assessment was not performed.

### Measure

The PEF assesses executive functions, including one practice item (i.e., using telephone) and 13 test items (i.e., sorting garbage, filling out deposit slip, buying necessities, using electric stove, diet control, withdrawing money, shopping under budget, using microwave, medicine management, using bus route map, paying bill, using street map, and addressing envelope) [9]. For each item, there are three instructions to assess the four domains (i.e., volition, planning, purposive action, and effective performance). In the first instruction for assessing the volition domain, an examinee is asked what he/she would do for a task in a provided item context and is rated on whether he/she could form a suitable goal. In the second instruction for assessing the planning domain, the examinee is asked how he/she would perform the tasks and is rated on whether he/she could recognize and organize the materials or steps to accomplish a goal. In the third instruction for assessing the other two domains, the examinee is asked to actually execute the task. In the purposive action domain, the examinee is rated on whether he/she executed the sequence of steps for the task. In the effective performance domain, the examinee is rated on whether he/she monitored and self-corrected the mistakes. We have presented an example for the sorting garbage item. The first instruction is “If you want to be environmentally friendly and you have a pile of garbage, what would you do?” The second instruction is “Have you ever sorted garbage? If you have a pile of garbage and need to sort them, how would you do it?” The third instruction is “Now you have to do a task. Please sort the garbage according to the garbage sorting sheet. After sorting, please give me the plastic bag. You can start when I say go. Do it as quickly as possible. Go!” Each domain is rated on a 3-point scale (0–2). The scoring criteria of the volition domain is 0 = no response or response not related to item context; 1 = response related to partial item context or not forming a suitable goal; and 2 = response related to complete item context and forming a suitable goal. The scoring criteria of the planning domain is 0 = no response or response not related to item context; 1 = response related to partial item context; and 2 = response related to complete item context. The scoring criteria of the purposive action domain is 0 = no action or doing one essential step of the task; 1 = doing ≥2 essential steps of the task; and 2 = doing all essential steps of the task. The scoring criteria of the effective performance domain is 0 = no action or making ≥2 mistakes; 1 = making one mistake; and 2 = not making mistakes or making mistakes but self-correcting the mistakes. The raw score of each domain is summed up as scores of 13 test items, ranged from 0 to 26. Based on the previous study, the PEF fits the assumptions of the Rasch model and thus, the ordinal scale of the PEF could be transformed into an interval-level scale [9]. Therefore, in this study, the raw score of each domain was transformed into a Rasch score and then linearly converted to a score ranging from 0 to 100, namely the Rasch transformed score (Appendix A). The Rasch transformed score was used for analysis in this study. The higher the score for each domain, the better the particular executive function that is targeted [9].

### Data analysis

We used intraclass correlation coefficient (ICC) to examine the consistency of the result of two assessments. The ICC was calculated based on an absolute agreement type under a two-way mixed model. An ICC value < 0.50 indicates poor reliability; 0.50–0.75 indicates moderate reliability; 0.75–0.90 indicates good reliability; and > 0.90 indicates excellent reliability [21]. An ICC value ≥0.80 indicates good reliability for group comparisons and ≥ 0.90 for individual comparisons [22]. For evaluating the heterogeneity of the data, the ICC values were calculated according to gender. The percentage of agreement of each item in the PEF was estimated. We computed the MDC at a 95% confidence level based on the ICC and standard error of measurement (SEM). The calculation formula of the SEM and MDC runs as follows [23].

SEM = SD × √(1 − ICC) (Formula 1).

MDC = 1.96 × √2 × SEM (Formula 2).

SD in formula 1 is the standard deviation of all scores in two assessments.

The study calculated MDC% to confirm whether the amount of random measurement error range was acceptable. The calculation formula of MDC% is: (MDC/highest possible score) × 100 [18]. If MDC% was less than 30%, then it was regarded as an acceptable random measurement error [24].

The paired *t*-test was used to evaluate systematic bias for examining whether a statistically significant difference is displayed between two assessments (two-tailed, α = 0.05). The Bland-Altman plot with 95% limits of agreement (LOA) was applied to visualize the agreement between two assessments: the differences of the two assessment were plotted against the mean of both assessments. The LOA was computed as a mean difference ± 1.96 × SD of the difference [25]. The plot allows one to observe whether tendency (heteroscedasticity) exists; for example, the differences between the two assessments generally increased when the mean values of both assessments increased [26]. Pearson’s *r* was used to analyze heteroscedasticity between the absolute differences and mean values of the two assessments. The data represented heteroscedasticity with *r* > 0.30 [27].

## Results

Each sample with 60 eligible people with schizophrenia participated in the intra-rater agreement study and inter-rater agreement study. The participants of the intra-rater agreement study had a mean age of 40.0 years, 46.7% were men, and the mean age at first onset was 22.6 years. The participants of the inter-rater agreement study had a mean age of 42.8 years, 43.3% of them were men, and the mean age at first onset was 21.2 years. Table 1 lists the detailed demographic information of the participants.

**Table 1:** Demographic information of participants

Variable	Intra-rater (*n* = 60)	Inter-rater (n = 60)
Age (mean year [SD])	40 (9.9)	42.8 (11.0)
Gender, n (%)		
Male	28 (46.7)	26 (43.3)
Female	32 (53.3)	34 (56.7)
Education, n (%)		
Elementary school	5 (8.3)	3 (5.0)
Junior high school	6 (10.0)	6 (10.0)
Senior high school	30 (50.0)	30 (50.0)
College and above	19 (26.7)	21 (35.0)
Age of onset (mean year [SD])	22.6 (7.4)	21.2 (7.1)
Type of antipsychotics, n (%)		
First generation	13 (21.7)	17 (28.3)
Second generation	50 (83.3)	49 (81.7)
Third generation	4 (6.7)	4 (6.7)
Taking two types of antipsychotics	7 (11.7)	10 (16.7)

*SD* standard deviation

Table 2 displays the results of intra-rater and inter-rater agreements of the test-retest reliability of the PEF. The ICC values of the four domains of the PEF were 0.88–0.92 for the intra-rater agreement study and were 0.82–0.89 for the inter-rater study. The ICC results of males (females) in the intra-rater agreement study for the volition, planning, purposive action, and effective performance domains were 0.93 (0.91), 0.94 (0.87), 0.87 (0.89), and 0.82 (0.94), respectively. The ICC results of males (females) in the inter-rater agreement study for the volition, planning, purposive action, and effective perofrmance domains were 0.85 (0.82), 0.82 (0.83), 0.76 (0.86), and 0.89 (0.88), respectively. The percentage of agreement of all items was 51.7–81.7 and 40.0%–81.7% in the intra-rater agreement study and inter-rater agreement study, respectively (Appendix B). The MDC values of the volition, planning, purposive action, and effective performance domains in the intra-rater agreement study (inter-rater agreement study) were 13.0 (15.8), 12.2 (17.4), 16.2 (20.9), and 16.3 (18.6), respectively. For the intra-rater and inter-rater agreement studies, the MDC% of the four domains in the PEF was < 30.0% (13.0–20.9%).

**Table 2:** Results of intra-rater and inter-rater agreements of the test-retest reliability of the PEF

Variable	Domain	Mean_1_ (SD_1_)	Mean_2_ (SD_2_)	ICC (95%CI)	MDC (MDC%)	Correlation^a^	*t* value (*p* value)
Intra-rater (*n* = 60)	Volition	59.4 (16.8)	60.0 (16.2)	0.92 (0.87,0.95)	13.0 (13.0%)	0.10	0.69 (0.490)
	Planning	47.2 (14.8)	47.4 (14.1)	0.91 (0.85,0.94)	12.2 (16.4%)	0.11	0.23 (0.822)
	Purposive action	58.2 (16.7)	62.2 (17.2)	0.88 (0.75,0.94)	16.2 (16.2%)	0.07	4.21 (0.000)*
	Effective performance	46.7 (18.3)	49.4 (18.2)	0.90 (0.82,0.94)	16.3 (18.8%)	0.01	2.67 (0.010)*
Inter-rater (*n* = 60)	Volition	58.9 (14.7)	61.5 (13.7)	0.84 (0.73,0.90)	15.8 (15.8%)	0.14	2.63 (0.011)*
	Planning	47.2 (16.4)	48.2 (13.9)	0.83 (0.73,0.89)	17.4 (20.0%)	0.29	0.92 (0.359)
	Purposive action	59.7 (17.2)	63.2 (18.5)	0.82 (0.71,0.89)	20.9 (20.9%)	0.13	2.61 (0.011)*
	Effective performance	48.0 (18.7)	50.4 (21.0)	0.89 (0.81,0.93)	18.6 (18.6%)	0.25	1.98 (0.052)

*PEF* Performance-based measure of Executive Functions, *SD* standard deviation, *ICC* intraclass correlation coefficient, *CI* confidence interval, *MDC* minimal detectable change

^a^Correlations between absolute differences and mean scores of the two assessments

*p < 0.05

In addition, for future reference, we have re-analyzed the ICCs, MDCs, and MDC%s of the PEF using raw scores, and the results were not dramatically different from the Rasch transformed data. We have provided the results of raw scores of the ICCs, MDCs, and MDC%s in Appendix C.

**Fig. 1:**
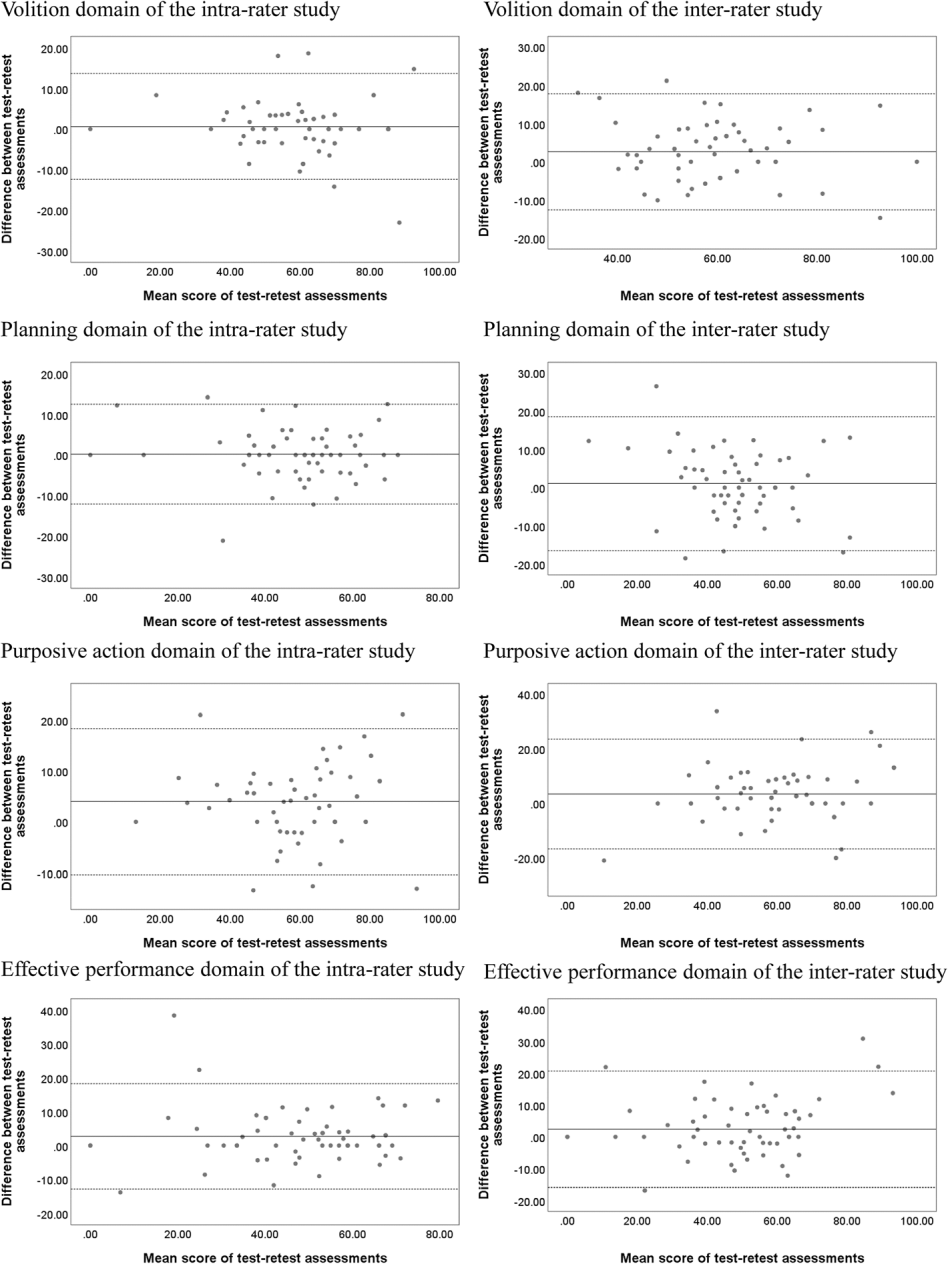
Bland-Altman plots. The bold line defines the mean difference and the two dotted line define the 95% limits of agreement

The results of the paired-*t* test showed that the purposive action and effective performance domains in the intra-rater agreement study and the volition and purposive action domains in the inter-rater agreement study showed statistically significant differences between two assessments (*p* < 0.05) (Table 2). The Bland-Altman plots of the four domains in the two samples are in Fig. 1. The LOAs in the intra-rater agreement sample were [− 12.4, 13.6] for volition, [− 12.1, 12.5] for planning, [− 10.4, 18.4] for purposive action, and [− 12.8, 18.3] for effective performance. The LOAs in the inter-rater agreement sample were [− 12.6, 17.8] for volition, [− 16.5, 18.6] for planning, [− 16.7, 23.6] for purposive action, and [− 15.9, 20.7] for effective performance. For the four domains, heteroscedasticity was not noticeable (*r* = 0.01–0.29) in the intra-rater and inter-rater agreement samples.

## Discussion

The ICC values of this study revealed good to excellent test-retest reliability, including intra-rater and inter-rater agreements. The ICC values of the four domains measured by the same rater (intra-rater agreement) or different raters (inter-rater agreement) were greater than the judgement standard for group comparisons (> 0.80). In other words, no matter whether the PEF was administered by the same or different raters, it displayed good consistent results for group comparison research. The ICC value of three domains (volition, planning, and effective performance) in the intra-rater agreement sample was ≥0.90, indicating that these domains can be used to assess scores for individuals with schizophrenia by the same rater. The four domains’ MDC% was < 30% in the intra-rater and inter-rater agreement studies, demonstrating that these domains have acceptable measurement errors by the same and different raters. According to our results, the PEF exhibited good test-retest reliability in people with schizophrenia. The PEF can be used to develop treatment plans and evaluate treatment effects in the initial assessment and follow-up assessments of outcome studies. Moreover, it has four domains (i.e., volition, planning, purposive action and effective performance) which can assess executive functions in a comprehensive manner.

The ICC values obtained by the same rater in each domain were higher than those by different raters, especially the volition and planning domains. The volition and planning domains measure participants’ thinking. The rater subjectively assesses the participants’ scores of these two domains based on their verbal responses. Appropriate answers for the volition and planning domains are provided in the manual of the PEF. However, not every participant’s response was provided in the manual, which can affect raters’ scores. In contrast, the purposive action and effective performance domains measure participants’ doing. Raters observed whether participants performed the necessary task steps. There are definite task steps for scoring in these two domains. Thus, the ICC values in the purposive action and effective performance domains were resemble between the same and different raters. Moreover, MDC% of the PEF domains in the inter-rater agreement study was relative higher than those in the intra-rater agreement study. That is, a relatively larger random measurement error was noticed between both raters. Due to difficulty in scoring the volition and planning domains and a larger random measure error, we recommend that future raters should receive rigorous training to improve test-test reliability administered by different raters.

The MDC values of the four domains in the PEF for the same rater and different raters are provided in this study. The implication of the MDC value is that when the score change between two consecutive assessments on the same individual is greater than the MDC value, there is a 95% confidence level to claim that the score change is beyond the random measurement error. The score change (>MDC) of the individual can be thought as real by users. Taking the volition domain of the same rater as an example (MDC = 13.0), when the score change between two assessments for a person with schizophrenia administered by the same rater is > 13.0, we can state that this person reveals real improvement in volition. The MDC values computed in this study can help clinicians and researchers to determine whether individuals’ volition, planning, purposive action, and effective performance conditions show real change after treatment under the same rater’s or different raters’ assessments.

In the intra-rater agreement study, two doing domains (i.e., purposive action and effective performance) showed revealed systematic bias. These two doing domains of the inter-rater agreement study also have relatively lower *p*-values (0.052–0.011). A possible reason is that in these two domains, participants needed to actually perform tasks and then make impressions on the task, resulting in systematic bias (e.g., practice effect) [28]. The volition domain showed a statistically significant difference in the inter-rater agreement study, but not in the intra-rater agreement study. The test order was fixed in the inter-rater agreement study (first and second assessments were conducted by raters A and B, respectively), which may have caused systematic measurement bias. Future studies are warranted to select a rater randomly from a rater pool to conduct the PEF in two assessments in order to examine systematic measure bias.

The Bland-Altman plot provides a visual evaluation of the degree of agreement between two assessments (e.g., identification of outliers of the two dotted lines and the correlation between the mean and variance of the assessment scores) [29]. In the Bland-Altman plots, the width of 95% LOA was wider in the four domains of the inter-rater agreement study, which may be due to the time window of 2 weeks between assessments. The correlation results displayed no heteroscedasticity for the four domains in the intra-rater and inter-rater agreement studies, demonstrating that the difference between two adjacent assessments did not change as the mean values of the two assessments increased. Our findings support that the fixed MDC value of each PEF domain estimated in the intra-rater and inter-rater agreement studies can be used for different levels of executive functions in people with schizophrenia.

This study has two limitations. First, this study used two convenience samples, which may limit the generalization of our findings. Additional studies with other samples with schizophrenia are needed to cross-validate our findings. Second, the ecological validity of the PEF has not been examined in people with schizophrenia, which restricts its use in explaining the results of executive functions in daily functions. Future studies need to examine the ecological validity of the PEF.

## Conclusions

The PEF showed sufficient test-retest reliability, including intra-rater and inter-rater agreements, for people with schizophrenia. The PEF can be reliably administered after brief instructions and training to assess and follow-up executive functions in people with schizophrenia.

## Supplementary Information

**Additional file 1.**

**Additional file 2.**

**Additional file 3.**

## Data Availability

The data and materials of the study are not publicly available, but are available from the third author on reasonable request.
