# Supplementary material for: Reliability of the performance-based measure of executive functions in people with schizophrenia
Source: BMC Psychiatry. 2021 Nov 10;21:553. doi: 10.1186/s12888-021-03562-y (PMC8579687; doi:10.1186/s12888-021-03562-y)
Supplement: Supplementary file 1 — Additional file 1. [file 12888_2021_3562_MOESM1_ESM.docx]

Appendix A. Raw score, logic score, and Rasch transformed score of the PEF

|  | Volition | | Planning | | Purposive action | | Effective performance | | |
| --- | --- | --- | --- | --- | --- | --- | --- | --- | --- |
| Raw score | Logic score | Rasch transformed score^*^ | Logic score | Rasch transformed score^*^ | Logic score | Rasch transformed score^*^ | Logic score | Rasch transformed score^*^ |  |
| 0 | -4 | 0 | -5.08 | 0 | -4.77 | 0 | -4.45 | 0 |  |
| 1 | -2.82 | 14.71 | -3.82 | 12.2 2 | -3.52 | 13.03 | -3.22 | 13.76 |  |
| 2 | -2.15 | 23.07 | -3.04 | 19.79 | -2.76 | 20.96 | -2.49 | 21.92 |  |
| 3 | -1.75 | 28.05 | -2.55 | 24.54 | -2.29 | 25.86 | -2.05 | 26. 85 |  |
| 4 | -1.46 | 31.67 | -2.18 | 28.13 | -1.93 | 29.61 | -1.72 | 30.54 |  |
| 5 | -1.23 | 34.54 | -1.86 | 31.23 | -1.64 | 32.64 | - 1.45 | 33.5 6 |  |
| 6 | -1.03 | 37.03 | -1.58 | J3.95 | -1.38 | 35.35 | -1.22 | 36.13 |  |
| 7 | -0.85 | 39.28 | -1.33 | 36.37 | -1.15 | 37.75 | -1.02 | 38.37 |  |
| 8 | -0.7 | 41.15 | -1.09 | 38.70 | -4.94 | 39.94 | -0.83 | 40.49 |  |
| 9 | -0.55 | 43.02 | -0.86 | 40.93 | -0.74 | 42.02 | -0.65 | 42.51 |  |
| 10 | -0.41 | 44.76 | -0.65 | 42.97 | -0.55 | 44.00 | -0.49 | 44.30 |  |
| 11 | -0.27 | 46.51 | -0.44 | 45.00 | -4.37 | 45.88 | -0.33 | 46.09 |  |
| 12 | -0.14 | 48.13 | -0.23 | 47.04 | -0.19 | 47.76 | -0.17 | 47. 87 |  |
| 13 | -0.01 | 49.75 | -0.02 | 49.08 | -0.01 | 49.64 | -0.01 | 49.66 |  |
| 14 | 0.12 | 51.37 | 0.18 | 51.02 | 0.17 | 51.51 | 0.14 | 51.34 |  |
| 15 | 0.26 | 53.12 | 0.39 | 53.06 | 0.35 | 53.39 | 0.3 | 53.13 |  |
| 16 | 0.39 | 54.74 | 0.6 | 55.09 | 0.53 | 55.27 | 0.47 | 55.03 |  |
| 17 | 0.54 | 56.61 | 0.82 | 57.23 | 0.73 | 57.35 | 0.64 | 56.94 |  |
| 18 | 0.69 | 58.48 | 1.05 | 59.46 | 0.93 | 59.44 | 0.82 | 58.95 |  |
| 19 | 0.85 | 60.47 | 1.29 | 61.78 | 1.14 | 61.63 | 1.01 | 61.07 |  |
| 20 | 1.03 | 62.72 | 1.56 | 64.40 | 1.38 | 64.13 | 1.22 | 63.42 |  |
| 21 | 1.23 | 65.21 | 1.85 | 67.22 | 1.64 | 66.84 | 1.46 | 66.11 |  |
| 22 | 1.47 | 68.21 | 2.18 | 70.42 | 1.94 | 69.97 | 1.73 | 69.13 |  |
| 23 | 1.76 | 71.82 | 2.58 | 74.30 | 2.31 | 73.83 | 2.07 | 72.93 |  |
| 24 | 2.17 | 76.93 | 3.11 | 79.44 | 2.79 | 78.83 | 2.52 | 77.96 |  |
| 25 | 2.84 | 85.29 | 3.93 | 87.39 | 3.56 | 86.86 | 3.26 | 86.24 |  |
| 26 | 4.02 | 100 | 5.23 | 100 | 4.82 | 100 | 4.49 | 100 |  |

^*^The logic score was linearly converted to a score ranging from 0-100.
