# Supplementary material for: Reliability of the performance-based measure of executive functions in people with schizophrenia
Source: BMC Psychiatry. 2021 Nov 10;21:553. doi: 10.1186/s12888-021-03562-y (PMC8579687; doi:10.1186/s12888-021-03562-y)
Supplement: Supplementary file 2 — Additional file 2. [file 12888_2021_3562_MOESM2_ESM.docx]

Appendix B. Percentage agreement of the PEF raw score

| Item | Domain | Intra-rater | Inter-rater |
| --- | --- | --- | --- |
| Sorting garbage | Volition | 68.3% | 76.7% |
|  | planning | 61.7% | 58.3% |
|  | purposive action | 68.3% | 65.0% |
|  | effective performance | 53.3% | 68.3% |
| Filling out deposit slip | Volition | 73.3% | 70.0% |
|  | planning | 58.3% | 58.3% |
|  | purposive action | 78.3% | 68.3% |
|  | effective performance | 71.7% | 65.0% |
| Buying necessities | Volition | 63.3% | 63.3% |
|  | planning | 63.3% | 55.0% |
|  | purposive action | 76.7% | 81.7% |
|  | effective performance | 60.0% | 75.0% |
| Using electric stove | Volition | 60.0% | 55.0% |
|  | planning | 56.7% | 40.0% |
|  | purposive action | 61.7% | 66.7% |
|  | effective performance | 55.0% | 60.0% |
| Diet control | Volition | 63.3% | 55.0% |
|  | planning | 66.7% | 73.3% |
|  | purposive action | 65.0% | 60.0% |
|  | effective performance | 73.3% | 55.0% |
| Withdrawing money | Volition | 66.7% | 60.0% |
|  | planning | 60.0% | 61.7% |
|  | purposive action | 66.7% | 68.3% |
|  | effective performance | 65.0% | 60.0% |
| shopping under budget | Volition | 68.3% | 71.7% |
|  | planning | 58.3% | 43.3% |
|  | purposive action | 61.7% | 66.7% |
|  | effective performance | 60.0% | 70.0% |
| Using microwave | Volition | 80.0% | 60.0% |
|  | planning | 56.7% | 43.3% |
|  | purposive action | 78.3% | 78.3% |
|  | effective performance | 60.0% | 58.3% |
| Medicine management | Volition | 60.0% | 63.3% |
|  | planning | 60.0% | 61.7% |
|  | purposive action | 60.0% | 78.3% |
|  | effective performance | 51.7% | 80.0% |
| Using bus route map | Volition | 73.3% | 80.0% |
|  | planning | 70.0% | 66.7% |
|  | purposive action | 66.7% | 65.0% |
|  | effective performance | 78.3% | 63.3% |
| Paying bill | Volition | 81.7% | 80.0% |
|  | planning | 58.3% | 58.3% |
|  | purposive action | 78.3% | 80.0% |
|  | effective performance | 68.3% | 65.0% |
| Using street map | Volition | 53.3% | 66.7% |
|  | planning | 70.0% | 68.3% |
|  | purposive action | 66.7% | 68.3% |
|  | effective performance | 63.3% | 65.0% |
| Addressing envelope | Volition | 66.7% | 73.3% |
|  | planning | 70.0% | 55.0% |
|  | purposive action | 81.7% | 73.3% |
|  | effective performance | 61.7% | 70.0% |
