# Supplementary material for: Reliability of the performance-based measure of executive functions in people with schizophrenia
Source: BMC Psychiatry. 2021 Nov 10;21:553. doi: 10.1186/s12888-021-03562-y (PMC8579687; doi:10.1186/s12888-021-03562-y)
Supplement: Supplementary file 3 — Additional file 3. [file 12888_2021_3562_MOESM3_ESM.docx]

Appendix C. Results of intra-rater and inter-rater agreements of the test-retest reliability of the PEF raw score.

| Variable | Domain | ICC (95%CI) | MDC (MDC%) |
| --- | --- | --- | --- |
| Intra-rater agreement (n=60) | Volition | 0.92 (0.87,0.95) | 4.90 (18.8%) |
|  | Planning | 0.90 (0.84,0.94) | 4.89 (21.3%) |
|  | Purposive action | 0.91(0.83,0.95) | 5.30 (20.4%) |
|  | Effective performance | 0.93 (0.87,0.96) | 5.31 (21.2%) |
| Inter-rater agreement (n=60) | Volition | 0.81 (0.69,0.88) | 6.81 (26.2%) |
|  | Planning | 0.85 (0.76,0.91) | 6.39 (25.6%) |
|  | Purposive action | 0.89 (0.72,0.94) | 5.84 (22.5%) |
|  | Effective performance | 0.90 (0.84,0.94) | 6.48 (24.9%) |

PEF= Performance-based measure of Executive Functions; SD= standard deviation; ICC= intraclass correlation coefficient; CI= confidence interval; MDC= minimal detectable change.
